# Wearable radio-frequency sensing of respiratory rate, respiratory volume, and heart rate

**DOI:** 10.1038/s41746-020-0307-6

**Published:** 2020-07-28

**Authors:** Pragya Sharma, Xiaonan Hui, Jianlin Zhou, Thomas B. Conroy, Edwin C. Kan

**Affiliations:** School of Electrical and Computer Engineering, Cornell University, Ithaca, NY USA

**Keywords:** Signal processing, Electrical and electronic engineering, Biomedical engineering, Respiration

## Abstract

Many health diagnostic systems demand noninvasive sensing of respiratory rate, respiratory volume, and heart rate with high user comfort. Previous methods often require multiple sensors, including skin-touch electrodes, tension belts, or nearby off-the-body readers, and hence are uncomfortable or inconvenient. This paper presents an over-clothing wearable radio-frequency sensor study, conducted on 20 healthy participants (14 females) performing voluntary breathing exercises in various postures. Two prototype sensors were placed on the participants, one close to the heart and the other below the xiphoid process to couple to the motion from heart, lungs and diaphragm, by the near-field coherent sensing principle. We can achieve a satisfactory correlation of our sensor with the reference devices for the three vital signs: heart rate (*r* = 0.95), respiratory rate (*r* = 0.93) and respiratory volume (*r* = 0.84). We also detected voluntary breath-hold periods with an accuracy of 96%. Further, the participants performed a breathing exercise by contracting abdomen inwards while holding breath, leading to paradoxical outward thorax motion under the isovolumetric condition, which was detected with an accuracy of 83%.

## Introduction

In addition to the effective treatment of diseases, modern medicine has been extended towards an overall healthier living with improved day-to-day quality of life^1,2^ and integrated end-of-life care not restricted to clinical visits^3,4^. This requires novel solutions for noninvasive vital-sign sensing without skin contact that can be integrated effectively into various lifestyles over broad age-groups. Such a technique could potentially help patients of asthma, chronic obstructive pulmonary disease (COPD)^5^, and sleep apnea by continuous monitoring of heartbeat and respiration.

In clinical practices, pulmonary function tests are generally assessed by spirometry^6^ and physical examinations^7^. The latter might not require any specialized instrument but is limited by the experience of the physician and is not quantitative. Spirometry provides detailed parameters characterizing lung function but requires attentive participation of the patient with forced breathing maneuvers, and is thus not feasible for continuous long-term measurements, or patients suffering from asthma. Whole-body plethysmography^8^ is used to measure absolute air volume in the lungs and breathing resistances without extensive voluntary exercises but still requires some patient cooperation. This poses a challenge for respiratory disorder detection in patients that are unable to follow the instructions due to weakness, coma, or other cognitive failures. Other alternatives for respiratory monitoring^9,10^ require calibration for the volume estimate, including nasal probes^11^ for airflow information, and strain or inductance belts at thorax and abdomen^9,12^, which need to maintain reasonable tension in all breathing conditions to measure chest-wall motion. Local strain sensors^13^ are more comfortable, but require tight skin contact to measure chest-wall motion. Gold-standard heartbeat monitoring uses electrocardiogram (ECG) with multiple adhesive skin-contact electrodes to monitor the electrical activity of the cardiac muscle. Pulse oximetry is a less invasive method of extracting the heartbeat, however, it requires a stable probe in direct contact with the skin^14–16^. Other noninvasive and non-skin-touch sensing technologies have been proposed, but also with their limitations. Thoracic surface and body vibrations due to heartbeat have been studied in detail using seismocardiography (SCG)^17–19^ and ballistocardiography (BCG)^17,20–22^, respectively, for signatures under different abnormalities. Both sensors measure either displacement, velocity, or acceleration originated from the heartbeat but observed at the body surface. Film-based displacement sensors may be affected by artifacts from structural vibrations in a moving wheelchair^23^ or ambulance. Integrated solutions like smart shirts^24^ with ECG, chest belts, and accelerometers can provide detailed cardiopulmonary characteristics, but have a large device form-factor, and are unsuitable for bedridden and geriatric patients where snug-fit clothing and skin-contact electrodes are impractical due to concerns of comfort and bed sores^25,26^. Ambient optical^27^ and radio-frequency (RF) sensing^28^ records surface motion from breathing and heartbeat with limited signal-to-noise ratio (SNR)^29,30^, and requires the reader to be in the line of sight (LoS) to the torso^31,32^. Far-field RF has additional limitations on the maximum number of subjects that can be measured simultaneously^33^. Respiratory volume (RV) is even more difficult to retrieve, as it is affected by body posture and orientation variation^32,34^ with respect to the antennas or cameras. Some noticeable work has been done using RF in the near-field region^35,36^ that can couple to internal dielectric boundary change to clearly measure respiratory motion, but heartbeat can only be extracted during breath-hold.

Near-field coherent sensing (NCS)^37^ is a noninvasive technique that works by transmitting a low-power continuous wave (CW) RF signal into the body with over-clothing antennas. The near-field coupling to the internal dielectric boundary motion results in a direct measurement of the heart, lung, and diaphragm motion, in contrast to surface motion sensors. Detailed high-frequency heartbeat characteristics associated with the S1 and S2 sounds can also be potentially extracted from over-clothing placement^38^. However, a detailed comparison with echocardiogram can help clarify this observation, while a further improvement from possible sensor fusion with SCG and phonocardiogram (PCG) needs to be investigated in future studies. The wearable RF sensor design has high ambient motion tolerance with the receiver placed close to the transmitter, making NCS less affected by environmental changes compared to direct far-field reflection^39^, where ambient motion within the antenna radiation pattern can cause significant interference. Multiple sensors can be placed on the body to couple to both respiratory and heartbeat motion with frequency multiplexing and can be easily extended to monitor multiple people in the same room. Owing to its simple transceiver architecture, this sensor can be readily designed in a small, convenient form factor, as well as made wireless-capable at low cost.

In our earlier work, we have demonstrated preliminary NCS results where RV and respiratory rate (RR) can be estimated with high accuracy for one participant in sitting posture^40^. In this paper, we present a setup to accurately estimate the heart rate (HR) and respiratory effort, including RR and RV, over 20 participants with varying body mass index (BMI) and gender. Further, as RV is sensitive to body posture and breathing styles, we have presented analyses over various postures, as well as during conscious and spontaneous breathing exercises with a large breathing range of 0–45 breaths per minute (BPM) and a resting HR in the range of 50–90 beats per minute (BPM). A short calibration period by a gold-standard pneumotachometer (PTM) was performed for each subject and posture once, and the corresponding model was used on NCS and chest belts for further voluntary breathing exercises.

## Results

### NCS sensor and test system architecture

The NCS sensing approach is based on the near-field coupling of ultra-high frequency (UHF) waves with the nearby dielectric boundary motion. The dielectric composition in the near-field region of an antenna will modulate its characteristics. For a single antenna, this change can be measured from the antenna reflection parameter *S*_11_. For an antenna pair, this can be derived from the cross-coupling *S*_21_. As the transmitter (Tx) and receiver (Rx) signal chains are better isolated in the *S*_21_ measurements with less self-interference and higher SNR, we opt to place an antenna pair as part of the NCS sensor to the region of interest, where the intended surface and internal boundary motion can be retrieved after baseband demodulation. Notice that UHF has reasonable penetration into dielectrics in the near-field, and thus the internal dielectric motion during breathing and heartbeat can be locally modulated onto the specific antenna pair. We have placed two NCS sensors on the chest, one below the xiphoid process close to the diaphragm, and another near the heart. These sensors measure the abdomen and thorax respiratory motion as well as the heartbeat, by effectively capturing the geometrical changes in these organs along with other associated muscles.

**Fig. 1:**
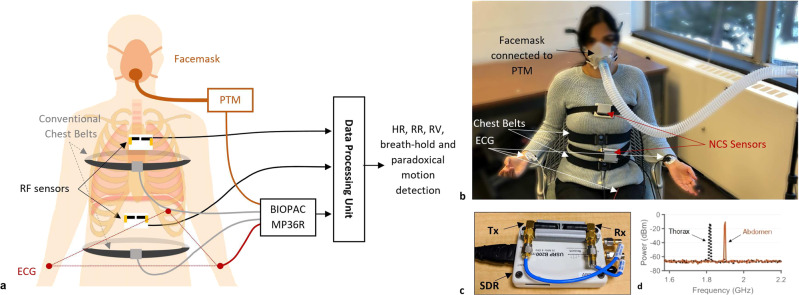
The experimental system. **a** Schematics of NCS and BIOPAC sensors and data flow. **b** Experimental setup with the participant wearing NCS and BIOPAC sensors in the sitting posture. The photo was taken and published with the written informed consent of the subject. **c** The NCS sensor consisting of SDR as well as the Tx and Rx antennas in a 3D-printed package. **d** Spectrogram of thorax and abdomen NCS sensors at their respective carrier frequencies of 1.82 GHz (−12.84 dBm) and 1.9 GHz (−10.42 dBm).

Figure 1a shows the NCS system architecture with sensor placement, data collection and processing flow. It also shows the reference sensors by BIOPAC (BIOPAC Systems, Inc., Goleta, CA), including ECG, chest belts and PTM connected to a facemask. A participant wearing all sensors in the sitting posture is shown in Fig. 1b. Two NCS sensors are held on the body with loosely placed belts. The abdomen sensor is placed slightly below the xiphoid process to be close to the diaphragm, and the thorax sensor close to the heart, so that it can couple both heartbeat and lung motion. These lightweight and small sensing units consist of RF Tx and Rx antennas connected to the software-defined radio (SDR) transceiver (Fig. 1c) to perform NCS measurements with a low RF power of less than —10 dBm (Fig. 1d), well under the safety limits set by occupational safety and health administration (OSHA)^41^.

**Fig. 2:**
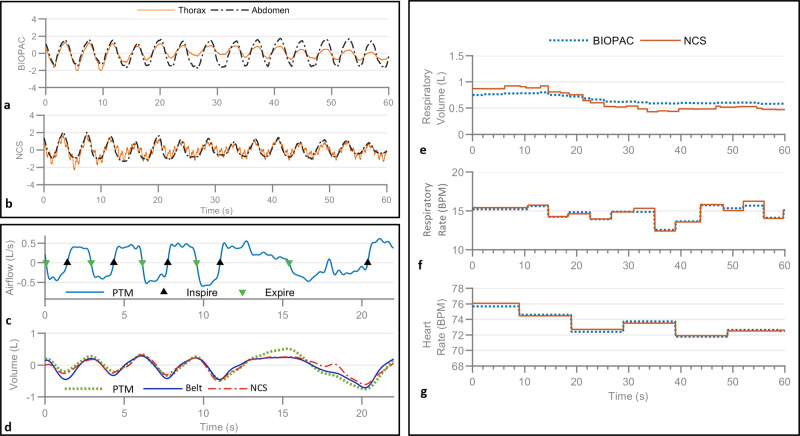
Examples of the sensor outputs and estimated RV, RR, and HR. **a**, **b** The normalized BIOPAC chest-belt signals (**a**) and normalized unfiltered NCS signals (**b**) showing respiration waveforms during tidal (normal) breathing. The NCS thorax signal shows strong heartbeat motion as well. **c** The PTM airflow signal with detected inspiration and expiration start points. **d** The extracted instantaneous volume ${\mathrm{Vol}}_{{\mathrm{PTM}}}$ (dotted green) from the airflow, and calibrated chest belts (blue) and NCS (dashed-dotted red) respiration volume waveforms. **e**–**g** The analysis of the data shown in **a** and **b**; **e** and **f** show RV and RR, respectively, from NCS and BIOPAC chest belts; **g** shows the HR from NCS and BIOPAC ECG.

The acquired BIOPAC and NCS signals are processed to estimate RV, RR, and HR statistics. The normalized BIOPAC thorax and abdomen chest-belt signals are shown as reference in Fig. 2a. Figure 2b shows normalized unfiltered NCS data from thorax and abdomen sensors, with strong respiratory motion modulation. Heartbeat is also modulated clearly on the NCS thorax waveform and can be properly filtered to remove respiratory motion. Supplementary Fig. 1 shows the filtered heartbeat waveform from NCS and the HR extraction techniques under motion artifact.

### RV calibration model

A calibration model is developed to estimate RV from NCS and chest belts in various postures and breathing conditions using airflow estimates from the pre-calibrated PTM connected to a facemask (Fig. 1b). While PTM is a kind of spirometer, we have referred to it as such to state explicitly that the airflow is estimated from the pressure change. The facemask design includes a separator between nose and mouth and is tightly strapped on to minimize any air leakage from the mouth. Participants are requested to inhale and exhale by mouth only, and they can feel rather uncomfortable during PTM measurements. Thus, PTM is removed from the participant except during the short initial calibration routine of 30 s, with 15 s of each normal and deep breathing. Instantaneous volume is computed by integrating the airflow rate for each breath cycle to get Vol_PTM_. Waveforms from both BIOPAC chest belts and filtered NCS respiration waveforms are then calibrated by the least-square fitting of linear relation:

1 $$a\cdot {\mathrm{Resp}}_{{\mathrm{Th}}} + b\cdot {\mathrm{Resp}}_{{\mathrm{Abd}}} + c = {\mathrm{Vol}}_{{\mathrm{PTM}}},$$

where Resp_Th_ and Resp_Abd_ are thorax and abdomen respiratory waveforms, respectively. Figure 2c shows PTM airflow waveform during the calibration, with the corresponding Vol_PTM_ and calibrated NCS and chest-belt volumes in Fig. 2d. RV is defined as the volume of the air exchanged during each inhalation and exhalation and is calculated as the peak height of each breath cycle. Figure 2e shows estimated RV from chest belts and NCS data shown in Fig. 2a, b, which have similar trends but NCS has shown more minute variations. This is consistent with Fig. 2d around 18 s, where the small perturbation in airflow is captured by NCS but not chest belts.

For RV calibration, as the posture can affect the breathing pattern and lung capacity, separate calibration is necessary^12^ for supine, lateral recumbent, and sitting postures for each participant. As the chest circumference has a significant variation across different postures, BIOPAC chest-belt tension requires re-adjustment to accurately measure respiration. The NCS sensor, on the other hand, only requires stable placement in the near-field region and does not require changes across different postures. Furthermore, during the sitting posture, we performed two more experiments with participants in relaxation and attention-task states without additional calibration. Frequent involuntary posture changes were observed during both states, either for comfort or in response to task failure or success. With these posture changes, tension-based BIOPAC belts inclined to move toward a stable, minimal-circumference position, i.e., lower for the thorax and upward for the abdomen belt, resulting in loss of tension and erroneous measurements. As the NCS belts and sensors were loosely placed, a posture change could cause the antennas to move closer or further away from the body, thus scaling the waveform amplitude. RV of BIOPAC and NCS during the known normal breathing sections were tested to apply a scaling correction. Detailed correction steps are discussed in the “Data Processing” section.

### Overall statistics

**Fig. 3:**
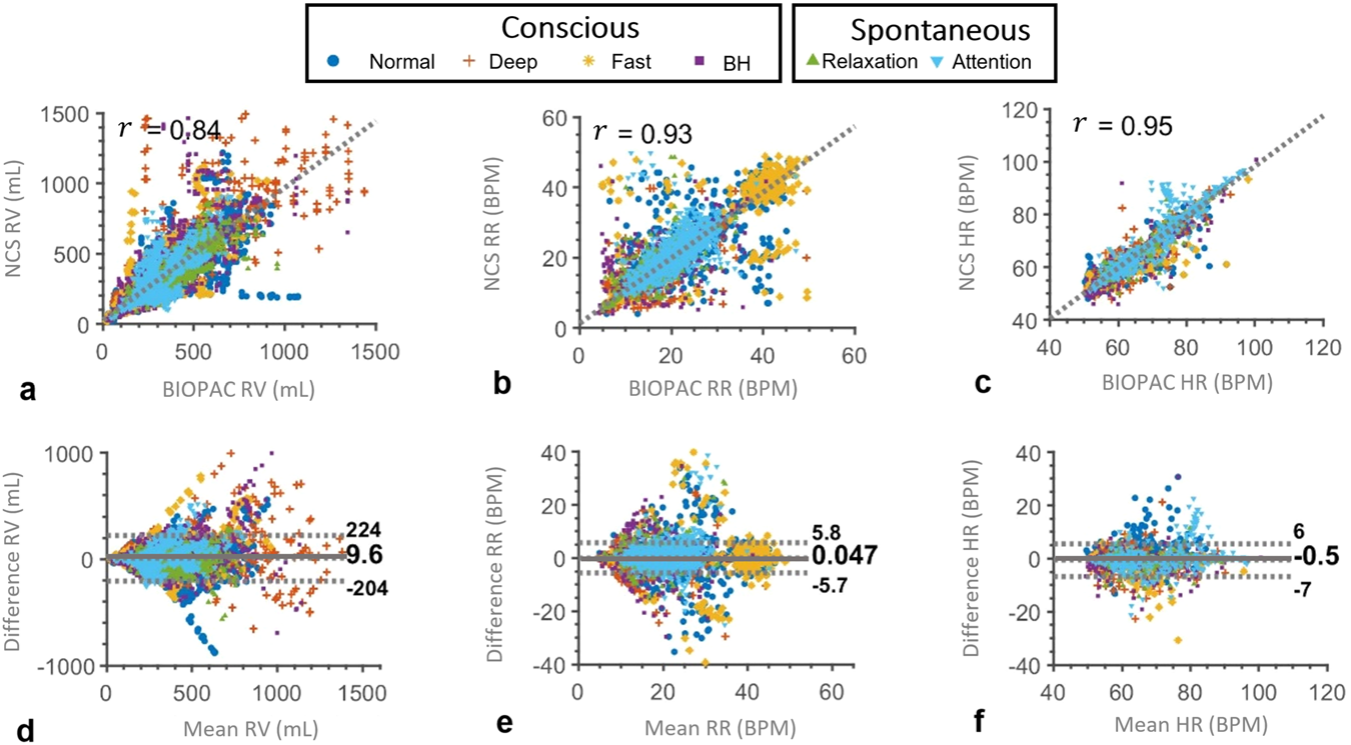
Correlation and agreement between NCS and BIOPAC estimate of RV, RR, and HR over the entire data. The label shows a marker for each breathing style, including conscious normal, deep, fast and breath-hold (BH), as well as spontaneous breathing in relaxation and attention states. **a**–**c** Scatter plots of NCS vs. BIOPAC RV, RR, and HR, respectively, with denoted Pearson’s correlation coefficient, $r$, showing a high correlation between the two sensors. **d**–**f**$B\& A$ plots of NCS and BIOPAC showing the bias $m$ at the center (solid line) and the corresponding LoA (dotted lines) given by $m \pm 1.96 \cdot \sigma$.

With tests in multiple postures and under different breathing exercises, we have performed analyses of 100 experiments, with over 590 min of total recorded data for 20 participants. Figure 3 shows the results across all three postures, with different breathing styles. We can achieve a high correlation of NCS and BIOPAC for RV $(r_{{\mathrm{RV}}} = 0.84)$, RR $(r_{{\mathrm{RR}}} = 0.93)$, and HR $(r_{{\mathrm{HR}}} = 0.95)$, as shown in the scatter plots in Fig. 3a–c. We have employed the Bland-Altman ($B\& A$) plot to quantify the agreement between NCS and BIOPAC, both of which may have errors. This agreement is estimated by the mean $(m)$ and standard deviation $(\sigma )$ of the measurement differences. $B\& A$ plots can also identify possible outliers visually by a $XY$ scatter plot, with the $Y$ axis as the pairwise difference, and the $X$ axis as the mean of the two measurements. The systematic bias is estimated as the mean difference $m$, and limits of agreement (LoA) within which 95% of the differences are expected to lie, are estimated as ${\mathrm{LoA}} = m \pm 1.96 \cdot \sigma$, assuming a normal distribution. Figure 3d–f show good agreement of both the sensors with low mean deviations ($m_{{\mathrm{RV}}} = 9.6$ mL, $m_{{\mathrm{RR}}} = 0.05$ BPM, $m_{{\mathrm{HR}}} = - 0.5$ BPM) and narrow LoA, as denoted by the dashed lines around the mean value. The results for each participant individually are shown in Supplementary Table 1.

### Variation over breathing patterns

As breathing is a hybrid voluntary-autonomous process, we have collected data during both conscious and spontaneous states. During conscious state, participants performed guided normal, deep, fast breathing and breath-holds. For the spontaneous tests, no breathing instructions were provided and instead, participants were first asked to relax and then execute an attention-engaging task. As performing the task required the participants to be seated, spontaneous breathing tests were performed only in the sitting posture. Figure 3 showed the results over all the experiments with breathing styles denoted by different markers. As normal breathing is similar to spontaneous breathing, most of these points are overlapped. The fast breathing pattern is centered around the instructed 40 BPM in the RR plots. Deep breathing is indicated by low RR around 6 BPM, as well as high RV. These points are also spread out with a low correlation between NCS and chest belts, possibly due to data distortion from the large motion, resulting in nonlinear strain gauge response, as well as NCS sensor motion relative to the body. Breath-hold periods have low RR at the beginning of the hold period and may also be accompanied with deep breaths after the long holding period, thus are seen closer to the deep-breathing points in both RV and RR plots. Zero RR is not marked, but these periods are treated separately during the breath-hold detection stage. Supplementary Fig. 2 shows an example of one participant following the conscious breathing protocol in the supine posture, and the extracted RV, RR and HR over time with consistent observations. Table 1a compares the correlation and $B\& A$ statistics for different breathing patterns. The relaxation state with spontaneous breathing shows the highest correlation of NCS and BIOPAC sensors for RV and HR estimates, close to conscious normal breathing results. This could be due to the regular breathing pattern with few motion artifacts. In comparison, participants tended to move in response to stress during the attention test, leading to motion artifacts.

**Table 1:** Correlation and $B\& A$ statistics.

Measurement		(a) Different breathing patterns						(b) Different postures with breathing protocol		
		Normal	Deep	Fast	Hold	Relaxation	Attention	Supine	Lateral recumbent	Sitting
RV (mL)	$r$	0.88	0.80	0.76	0.84	0.93	0.76	0.81	0.89	0.81
	$m$	0	60	18	33	−10	11	2	18	27
	$\sigma$	89	186	144	138	65	97	121	115	132
RR (BPM)	$r$	0.95	0.91	0.89	0.77	0.93	0.87	0.94	0.93	0.93
	$m$	0.05	0.02	−0.49	−0.13	0.11	0.19	−0.06	0.06	−0.08
	$\sigma$	2.48	2.58	6.66	4.09	1.72	2.48	2.84	3.60	3.52
HR (BPM)	$r$	0.95	0.93	0.90	0.96	0.98	0.95	0.96	0.93	0.94
	$m$	−0.69	−0.98	−1.70	−0.80	−0.23	0.04	−0.70	−0.62	−1.22
	$\sigma$	2.90	3.45	4.38	2.92	1.81	3.67	2.72	3.54	3.20

### Variation over postures

Adjusting body postures can lead to RV variations, as respiratory mechanisms are affected by different resistance or compliance of the lung and chest-wall components. Thus, we performed tests with the guided breathing protocol in three postures: laying on a bed in supine and left lateral recumbent postures and sitting in a chair. The average reference RV, calculated over all 20 participants during the identical protocol of voluntary breathing exercises, is observed to be highest at 329 mL in the supine posture, followed by sitting and lateral recumbent at 317 and 263 mL, respectively. The RR has the opposite trend with average RR of 17.3, 19, and 19.5 BPM in supine, sitting, and lateral recumbent postures, respectively. The average HR is highest during sitting at 67.6 BPM, compared to 65.3 and 63.8 in supine and lateral recumbent postures, respectively. These results are summarized in Supplementary Table 2. The detailed statistics for each posture are shown in Table 1b. Good correlation is observed across all the postures, with supine showing the least bias ($m$) for RV and RR estimates with narrow LoA (small $\sigma$), possibly due to the stable posture. No other clear trend is observed, indicating that the estimates are more sensitive to breathing types and patterns than to the posture. Supplementary Fig. 3 shows the correlation and $B\& A$ plots for different postures, both with and without the simulated breathing exercises.

### Breath-hold detection accuracy

Each participant was instructed to perform two breath-holds in both supine and lateral recumbent postures for a maximum duration of 20 s, simulating central sleep apnea (CSA), which is indicated by breath cessation for at least 10 s^42^. A simple detection algorithm was implemented based on the inspiration peak-to-peak interval. Overall, both NCS and BIOPAC performed well for breath-hold detection, as shown in Table 2, which shows both sensors detected 74 cases out of 80 annotated cases, with each missing 3 non-overlapping instances. The errors possibly originate from the participant’s incompliance to the breath-hold protocol. Small torso motion is coupled differently to the two sensors, leading to incorrect peak detection during breath-hold. Supplementary Fig. 4 shows the NCS and BIOPAC respiratory waveforms with detected peaks during breath-holds for representative good and poor cases.

**Table 2:** Detection of breath-hold (BH) and paradoxical abdomen-thorax (PAT) motion.

BH $(n = 80)$		NCS		PAT $(n = 58)$		NCS	
		Detected	Missed			Detected	Missed
BIOPAC	Detected	74	3	BIOPAC	Detected	42	9
	Missed	3	0		Missed	6	1

### Paradoxical abdomen-thorax motion detection accuracy

**Fig. 4:**
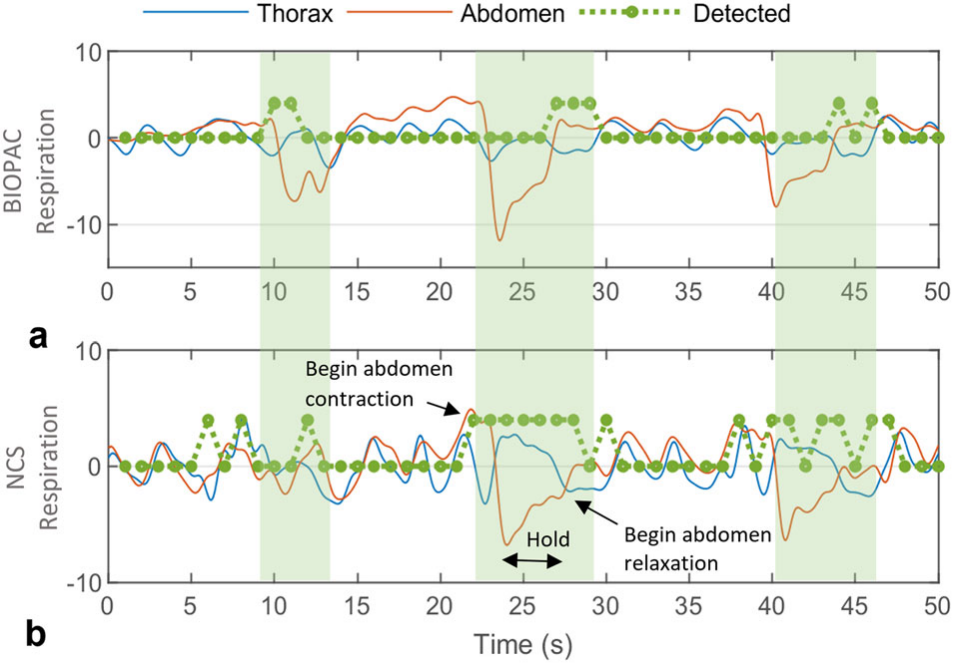
An example of normalized thorax and abdomen respiration waveforms during the isovolumetric maneuver by one participant. **a** Normalized BIOPAC chest-belt waveforms and **b** Filtered NCS respiration waveforms. The intended paradoxical motion windows are marked by green-shaded areas and detected instances are shown by the positive value of dotted green lines. Timing of abdomen contraction, hold, and relaxation is denoted during the second cycle of the NCS waveform. Both BIOPAC and NCS can detect all three instances of paradoxical abdomen-thorax motion.

To test the separate thorax and abdomen motion, the participants were asked to perform an isovolumetric abdomen exercise while holding breath. With no airflow, the inward abdomen contraction results in outward motion of the thorax, as the total lung volume is conserved^43^, simulating paradoxical abdomen-thorax motion similar to obstructive sleep apnea (OSA) with complete closure of the airway. We used the slope-product of thorax and abdomen respiration waveforms to detect paradoxical motion in BIOPAC and NCS waveforms, as shown in Fig. 4, where three instances of isovolumetric maneuver are successfully detected by both the sensors. While some participants were able to successfully perform breath-hold and abdomen contraction, anomalous instances when paradoxical behavior was not observed can be attributed to the following possible reasons. (1) Placement sensitivity of sensors: the BIOPAC thorax belt placed near the xiphoid process may be coupled to the abdomen motion, and similarly the NCS sensor can be coupled to the accessory muscles. Cross-coupling between the two motions will reduce the paradoxical motion detectability. (2) Participants were unable to perform the isovolumetric exercise correctly during the intended period while following the breath-hold constraint. (3) The signal is lost due to sensor instability during the large chest circumference change resulting from the abdomen contraction. Overall, the algorithm was designed to be able to detect even slight paradoxical motion, resulting in the similar performance of both sensors as shown in Table 2. Both NCS and BIOPAC can detect 42 out of 58 instances, with fewer missed cases for BIOPAC (6) than for NCS (9). Supplementary Fig. 5a, b show two examples of poor detection for NCS and BIOPAC, respectively. While the algorithm performs well, as seen in these figures, it is more sensitive to baseline drift and other motion artifacts. Thus, sensor placement needs to be further investigated for robust paradoxical motion recognition.

### Motion interference with NCS

Our wearable NCS sensor setup has a high tolerance for ambient motion interference. As shown in Supplementary Fig. 6, there is no interference from a nearby walking person on the NCS data, due to the strong near-field coupling. Additional tests are performed with the motion of different body parts during breath-holds, as shown in Supplementary Figs. 7 and 8. There is no effect of hand motion on BIOPAC sensors, nor the NCS sensors unless it is very close to the antenna pair. ECG becomes noisy with the hand motion, likely due to electromyogram (EMG) interference. During arm motion, both BIOPAC thorax and abdomen belts show signal anomalies, while only the thorax NCS is affected. Additional studies are required to examine internal muscle motion interference resulting from near-field coupling, and separate the external arm motion coupling, as abdomen NCS still shows clear signal without any interference.

While external motion causes limited interference, large spurious torso motion results in motion artifacts. Respiratory measurements from BIOPAC chest belts can be interfered with belt displacement due to body movement, and an entire signal segment can be missed if the participant performs heavy or forced exhalation that leads to loss of tension. NCS waveforms are prone to motion artifact due to the current antenna pair packaging, which is loosely placed over the clothes with semi-isotropic radiation patterns. Free antenna movement relative to the body surface may result in signal artifacts, making NCS well-suited for static and quasi-static conditions, for example, when a person is typing, driving, or sleeping.

As errors in either BIOPAC or NCS respiratory data will result in inconsistent RV and RR, which reduces the reported accuracy, data quality score is defined, where poor respiration data quality intervals are rejected. Similarly, poor heartbeat data is also discarded based on NCS data quality. Further quality estimation details are discussed in the “Data Processing” section.

## Discussion

Following the prescribed routines, the subjects performed breathing exercises with large RR variation, from breath-holds and slow deep-breathing around 6 BPM to panting around 40 BPM. Our sensor and algorithm can achieve high accuracy under these large variations, with no special clothing or environmental requirements, thus promoting the applicability in real-life conditions for continuous monitoring of healthy subjects. A brief comparison with other noninvasive techniques focused on respiration is presented in Table 3. Different statistics are reported, including percentage accuracy and root mean square error (RMSE), apart from the ones used in this paper, $r$, $m$, and $\sigma$. Our proposed NCS method provides a good estimation of both heart and respiratory motion characteristics with increased user comfort. The accuracy tolerance for a well-calibrated gold-standard spirometer is ±3%^6^. For example, a quiet breath of 0.4 L will be measured as 0.4$\pm$0.012 L, and a deep breath of 2 L will be measured as 2.0$\pm$0.060 L. Table 1a shows the $\sigma$ for different breathing patterns, with low values in spontaneous normal breathing conditions, $\sigma _{{\mathrm{Relax}}} = 0.065$ L, $\sigma _{{\mathrm{Attention}}} = 0.097$ L, and $\sigma _{{\mathrm{Normal}}} = 0.089$ L during the breathing protocol. While these values are higher than acceptable clinical values, they can be utilized for preliminary analysis where quantitative evaluation of respiratory efforts including both RR and RV along with HR is helpful, especially for geriatric patients with low cognitive function, where dyspnea information can only be currently determined from self-reporting or a caregiver’s visual observation^44,45^.

**Table 3:** Comparison with other noninvasive methods.

Paper	Sensor	Statistics			Experimental conditions
		Respiratory rate	Respiratory volume	Heart rate	
Massagram et al.^34^	Far-field doppler RF; 1 m LoS	–	Sitting: *r* = 0.77; $m$ = 39 mL; $\sigma$ = 107 mL Supine: *r* = 0.72; $m$ = 24 mL; $\sigma$ = 129 mL	–	• 8 healthy • spirometer
Nguyen et al.^32^	Directional far-field CW RF	–	Bed accuracy: 95.4% (error 58 mL)	–	• 6 healthy • spirometer
Adib et al.^33^	Far-field FMCW RF; 1–8 m LoS	Accuracy: 99.3% (error 0.09 BPM)	–	Accuracy: 98.5% (error 0.95 BPM)	• 14 healthy • chest-belt/oximeter
Reyes et al.^27^	Smartphone camera	$r$ = 1.0; RMSE = 0.4 BPM; *m* = −0.02 BPM; $\sigma$ = 0.42 BPM	$r$ = 0.98; RMSE = 182 mL; *σ* = 185 mL	–	• 15 healthy • spirometer
Brüllmann et al.^12^	RIP chest belts	–	Healthy: *m* = 0 mL; *σ* = 55 mL Patients: *m* = 20 mL;*σ* = 100 mL	–	• 5 healthy, 12 patients • flow meter
Chu et al.^13^	On-skin strain sensor	–	$r$ = 0.96; *m* = −77 mL; *σ* = 152 mL	–	• 7 healthy • spirometer
NCS^37,40^	Wearable RF	*r* = 0.93; *m* = 0.05 BPM; σ = 2.93 BPM	*r* = 0.84; *m* = 10 mL; *σ* = 109 mL	*r* = 0.95; *m* = −0.5 BPM; *σ* = 3.32 BPM	• 20 healthy • PTM

A comparison of the proposed NCS method (across all positions and breathing styles) with existing technologies, focused on respiration. Gold-standard spirometer respiratory volume accuracy tolerance is ±3%^6^. Different performance metrics are used across papers, including percentage accuracy, root mean square error (RMSE), correlation coefficient (*r*), mean (*m*) and standard deviation (*σ*) of the differences of measured and reference data (*B*&*A* statistics). Experimental conditions show number of participants and the reference measurement device for each work. The last row shows the results of the presented work.

*LoS* line of sight, *FMCW* frequency modulated continuous wave, *RIP* respiratory inductance plethysmography.

The limitations of the current setup include data distortion due to antenna motion relative to the body, sensor placement sensitivity and arduous calibration requirements. The former issue of antenna motion can be readily resolved by improved antenna packaging and garment integration of the antenna with conductive fibers^46^. While sensor placement requirements are less stringent for most routines, it becomes more important for accurate paradoxical abdomen-thorax motion monitoring, as NCS sensor coupling to diaphragm and lungs is observed to be position sensitive and needs to be carefully deployed. Our calibration protocol requires participants to breathe only through the mouth while wearing a facemask for a brief duration. This calibration is mainly to give an absolute scale in RV and can be omitted if the percentage volume change is sufficient.

Additionally, the NCS sensor with its small form-factor and simple transceiver architecture can be incorporated inconspicuously into the fabric with an improved wireless design and can be worn over multiple layers of clothing without requiring any direct skin contact. We have used frequency division for multiplexing two sensors, which can be easily extended to place more sensors at different locations on the body, as well as on multiple people simultaneously. The Tx signal can be further modulated with a unique pseudo-noise code known to the corresponding Rx. These design options in NCS provide higher signal isolation against ambient interferences and inter-sensor collision than implementations based on the direct far-field RF and optical sensors. The detailed respiratory and heartbeat characteristics also open other areas of applications, including cough monitoring, stress detection, and overall ambulatory healthcare monitoring.

## Methods

### Hardware setup

The NCS sensor prototype is implemented by an Ettus universal software radio peripheral (USRP) B200mini^47^ SDR, with monopole helical antennas (Taoglas TG.19.0112), packaged in a three-dimensional-printed case, as shown in Fig. 1c. The relative antenna placement is designed to adjust the direct Tx-Rx coupling and enhance the sensitivity to the reflected signal from internal organs. Two SDR units are multiplexed by frequency, with the carrier frequencies of 1.82 and 1.90 GHz. This setup is a variation of the previous multiple-input multiple-output (MIMO) implementation^40^ to improve sensor stability and participant comfort. The baseband tone is set at $f_{\mathrm{BB}}$ = 51 kHz, sampled at $2 \times 10^6$ samples per second (Sps), where the quadrature baseband signals $I\left( t \right)$ and $Q\left( t \right)$ are simple sinusoidal tones. The intended motion is modulated on the IQ amplitude (${\mathrm{NCS}}_{{\mathrm{amp}}}$) and phase (${\mathrm{NCS}}_{{\mathrm{ph}}}$), and can be extracted as,

2 $${\mathrm{NCS}}_{{\mathrm{amp}}}\left( t \right) = \sqrt {I_{{\mathrm{Rx}}}\left( t \right)^2 + Q_{{\mathrm{Rx}}}\left( t \right)^2} ,$$

3 $${\mathrm{NCS}}_{{\mathrm{ph}}}\left( t \right) = {\mathrm{unwrap}}\left( {{\mathrm{tan}}^{ - 1}\left( {{\textstyle{{Q_{{\mathrm{Rx}}}\left( t \right)} \over {I_{{\mathrm{Rx}}}\left( t \right)}}}} \right) - 2\pi f_{{\mathrm{BB}}}t + \theta _0} \right),$$

where $\theta _0$ is a constant phase offset including the initial phase and accumulation from the Tx-Rx path and cables. While both amplitude and phase contain the modulated motion, their coupling strength varies with the sensor placement, which provides further resistance to noises when both magnitude and phase are included for further analysis. Additional studies are needed to clearly understand the coupling tradeoffs. For this work, we opted to use the best reference-correlated signal during the calibration phase for both thorax and abdomen sensors. For respiration, chest-belt signals are taken as reference, and for heartbeat, bandpass filtered (0.9–1.8 Hz) ECG signal is taken as the reference. The final demodulated data is sent to the control computer by a universal serial bus. The Tx powers are $- 12.84$ dBm and $- 10.42$ dBm for the thorax and abdomen, respectively, as shown in Fig. 1d, significantly below the allowable OSHA radiation exposure limit^41^.

Reference measurements are performed by BIOPAC sensors, including a 3-lead ECG SS2LB, two torso belts SS5LB and PTM SS11LB, placed as shown in Fig. 1a, b. The pre-calibrated PTM measures the airflow rate in L/s from the mouth using a facemask and is only placed on the subject for short calibration periods. For the remaining breathing exercises, reference respiration is only recorded by two belts placed at thorax and abdomen that measure the change in local tension. During the experiment, male participants wore the thorax belt at 2–3 cm below the armpits, while female participants wore the belt below the breasts, close to the xiphoid process and costal margin, considered as the dividing line between the rib cage and abdomen. This placement is selected for belt stability and user comfort. All sensors are connected to a 4-channel data acquisition unit BIOPAC MP36R^48^.

### Participants and protocol

The human study protocol was approved by the Cornell Institutional Review Board (IRB), and participants provided written informed consent to take part in the study. Twenty-five healthy participants with no known history of cardiopulmonary diseases were recruited and instructed to follow breathing routines to the best of their abilities, without overexerting, in a sequence of three postures: supine, left lateral recumbent and sitting upright in a chair. The instructions were provided to the participants in real-time using LabVIEW in both audio and visual formats. The data collection was carried out in a standard laboratory room with drywalls and supporting metal frames, without any radiation-absorbent material. The environment consisted of standard furniture, including a bed, desks, and chairs, along with computers and various units of laboratory equipment. The participant attire was not controlled, and the NCS sensors were placed over their daily clothing, including shirts and loosely fitted hoodies made of different fabric materials. Five participants’ data was rejected due to poor calibration data in any one of the three postures, which showed inconsistency due to either an inability to follow the mouth-only breath instruction or from the loose placement of the facemask that caused air leakage.

The 20 eligible participants included 14 females and 6 males with age from 18 to 34-year-old ($\mu = 22.9,\,\sigma = 3.3$), weight from 49.8 to 79.5 kg (*μ* = 62.2, *σ* = 9.3) and height from 158 to 183 cm ($\mu = 167,\,\sigma = 6.8$). The body mass index (BMI) of the participants fell within the slightly underweight to slightly overweight range from 18.3 to 26.8 ${\mathrm{kg}}/{\mathrm{m}}^2$ ($\mu = 22.2,\,\sigma = 2.2$).

A fixed breathing protocol of ~6 min was executed in each posture after calibration, which included:

1. Normal breathing for 120 s.
2. Deep breathing for 60 s.
3. Fast breathing for 30 s.
4. Normal breathing for 30 s.
5. Breath-hold for maximum 20 s, followed by normal breathing for 20 s. Repeat once.

During normal or tidal breathing, no inhalation or exhalation instructions were provided, but the participants were asked not to take any deep breaths or long pauses. During deep breathing, real-time instructions were given to start inhaling and exhaling with fixed durations of 4 and 6 s, respectively, which gave a RR of 6 BPM. Similarly, instructions were given for fast breathing at a rate of 40 BPM, with equal time for inhalation and exhalation. These instructions were provided as a guideline, and participants were advised to perform normal breathing if they felt uncomfortable during any of the routines, resulting in some variations around the expected rates. The procedure was clearly explained to the participants, with short examples for practice, before recording the calibration and main routines.

For observing separate thorax and abdomen motion, the participants were asked to perform the isovolumetric exercise with the following steps during breath-hold: contract the abdomen inwards, maintain the position for 3 s, then relax abdomen back to normal. Three such maneuvers were performed, separated by normal breathing intervals of 10 s to simulate paradoxical abdomen-thorax motion as observed in OSA. As this motion is difficult to perform without extended practice, this exercise was only performed in the sitting posture, so that the participants could learn the motion, while looking at their real-time NCS waveforms.

With the participants continued to be seated in a chair, an additional series of exercises were performed to test the breathing patterns in the relaxation state and under the given attention-engaging task. The participants were asked to close their eyes and relax for 5 min. This relaxed period allowed free-breathing over time without any instructions, thus increasing the likelihood of non-voluntary tidal breathing. The next routine was designed using PsyToolkit^49,50^ to render the participants under an attentive and cognitive task, where they watched and reacted to anomalous jumps of an on-screen rotating clock hand by pressing the space bar. Instantaneous feedback was given by flashing an on-screen indicator light of correct keypress, to ensure continuous attention. This routine was performed for 6.5 min and rendered non-voluntary breathing patterns under fast temporal variations due to induced stress. Both routines utilized the RV calibration from the previous sitting posture.

### Data processing

The synchronization among NCS and BIOPAC sensors is achieved by system time stamps and improved by maximizing the correlation between respiration data from two measurement systems, resulting in synchronization on the order of hundreds of ms (<1 s). The data collected from both sensors is downsampled to 500 Sps before further analysis in MATLAB.

#### Peak detection

For peak detection, a moving average-crossing algorithm^51^ is implemented, which is effective for signals with varying amplitude and frequency characteristics, such as the respiration signal here with the RR in the range of 0–45 BPM, without any manual tuning. A moving average is estimated at every point using a given window length, resulting in a moving average curve (MAC). This window length is selected to have approximately one respiratory cycle in each window and is constantly updated by taking Fourier transform over a fixed period to estimate the RR. The points where MAC crosses the original signal are labeled as intercepts and are classified as up or down intercepts for positive and negative slopes, respectively. Finally, a maximum is marked as the maximal point between two up-down intercepts and a minimum is also marked similarly. This algorithm is used in the estimation of all the three parameters: RV, RR and HR.

#### RV estimation

PTM measures the airflow rate, from which the beginning of each inhalation and exhalation points are identified by a simple zero-cross detection algorithm, based on the change of sign before and after the zero-crossing points, and the slope at these points. Integration is performed over each inhalation and exhalation cycle to get the instantaneous air volume, Vol_PTM_, without aggregating a bias over time. Least-square fitting is performed for solving Eq. (1) by a trust-region algorithm with bounded constraints of *a*,*b* > 0. RV is estimated as the average volume exchanged in each respiratory cycle over a window of 15 s, with at least two peaks in the window to get a robust estimate.

Owing to sensor motion over time, mostly resulting from a posture change, the estimated RV may deviate from its original calibration. An additional corrective step is performed if the estimated RV during normal breathing section of the breathing exercise routine ${\mathrm{RV}}\left( {{\mathrm{Normal}}} \right)_{{\mathrm{Routine}}}$, deviates more than ±5% from the calibrated ${\mathrm{RV}}\left( {{\mathrm{Normal}}} \right)_{{\mathrm{Calibration}}}$, derived from the first 15 s of calibration. The NCS and BIOPAC RV is multiplied with a corresponding scaling factor (SF) given as ${\mathrm{RV}}\left( {{\mathrm{Normal}}} \right)_{{\mathrm{Calibration}}}/{\mathrm{RV}}\left( {{\mathrm{Normal}}} \right)_{{\mathrm{Routine}}}$, assuming the normal breathing routine has nearly constant RV for the same person over time. To validate this correction, an additional RV calibration consistency study was conducted on one subject over three consecutive days in the sitting posture. The subject performed the calibration, immediately followed by the voluntary breathing exercise routine. Supplementary Table 3 shows the detailed results, with RV before scaling correction, and the required SF to improve the match when using day-1 calibration for all successive days. The ${\mathrm{RV}}\left( {{\mathrm{Normal}}} \right)_{{\mathrm{Calibration}}}$ lies within a narrow range of 0.37–0.43 L, showing consistency in normal breathing volume for a healthy individual over time. Little scaling is required to correct RV when calibrated immediately before the routine, resulting in scaling factors close to 1 on day-1. Variations in sensor placement and coupling strength result in scaling factors other than 1 to correct the RV for the next two days. Supplementary Fig. 9a, b show instantaneous volume and RV estimated on day-2 using day-1 calibration, with NCS underestimating and BIOPAC overestimating the expected ${\mathrm{RV}}\left( {{\mathrm{Normal}}} \right)_{{\mathrm{Calibration}}}$ = 0.37 L, derived from day-1. The corrected scaled RV in Supplementary Fig. 9c shows improved agreement of NCS and BIOPAC to each other, as well as to the calibration value. As SF assumes accurate ${\mathrm{RV}}\left( {{\mathrm{Normal}}} \right)_{{\mathrm{Calibration}}}$, some error may be introduced from inconsistencies in the calibration and correction process, including variation due to small calibration duration, difference in resistance when breathing through the mouth, and change in ${\mathrm{RV}}\left( {{\mathrm{Normal}}} \right)$ over time. The correction assumes knowledge of normal breathing period, that can be potentially identified based on the normal RR and HR range of an individual.

We have also studied the influence of different simulated airway resistances on RV by introducing two resistances: (1) KN95 mask covering face and mouth while breathing normally through the nose, and (2) straw with a 4 mm inner diameter while only breathing through the mouth. The extracted instantaneous volume, RV and RR from chest belts and NCS are shown in Supplementary Fig. 10. RV calibration was performed before the entire routine. While breathing through mask does not show any noticeable change in RV and RR estimates, breathing through straw shows decreased RV towards the beginning with constant RR, followed by an increase in RV with decreased RR. This opposite RV and RR behavior indicate the increased respiratory effort required due to the resistance offered by the narrow straw. The consistency of RV between the NCS and BIOPAC suggests that the respective calibration remains reasonable within the tested range of varying simulated airway resistance.

#### RR estimation

The NCS signal is bandpass filtered with the cut-off frequencies of 0.05 and 0.8 Hz to derive the respiration waveforms from both the sensors. As the diaphragm and resulting abdomen motion is usually larger, the respiration waveform from the abdomen NCS sensor alone is used to estimate RR. This sensor also has a weaker heartbeat coupling, and thus filtering requirements are less stringent during fast breathing and breath-hold. Similarly, the BIOPAC abdomen belt is also used to estimate RR with the same algorithm. RR is calculated as the number of detected breath cycles over the maximum window of the past 15 s. For each window, the number of cycles is calculated as the number of inhalation peaks minus 1, and the total time is the interval between first and last inhalation peaks. If no complete cycle is detected during the entire window, the RR is marked as 0. Figure 2f shows calculated RR from NCS and BIOPAC during a normal breathing section, between 12–17 BPM. Supplementary Fig. 2d shows estimated RR over varying normal, deep and fast breathing and breath-holds, in the range of 0–45 BPM.

#### HR estimation

The heartbeat waveform is modulated strongly on the thorax sensor and can be separated from the respiration by proper filtering. However, during the fast breathing exercise, the RR can be around 40 BPM, close to the HR, resulting in filtering ambiguity. Therefore, we have used the second harmonic of the heartbeat waveform (Supplementary Fig. 1) to estimate the peak-to-peak heartbeat interval with reduced interferences from respiration and motion. The Fourier transform is used to first estimate the approximate HR, which guides the filtering to retrieve the second harmonic with a bandwidth of 0.8 Hz. The same peak detection method is then used to find peaks of the second-harmonic waveform. Instantaneous HR is estimated as an inverse of each heartbeat interval, taken as the sum of two neighboring peak-to-peak periods, as shown in Supplementary Fig. 1. This HR is averaged over a window of 10 s to suppress any outliers. The reference HR is estimated from the ECG waveform with bandpass filtering between 0.4 and 20 Hz, which removes any baseline drift and suppresses the low-frequency P and T waves. The sharp QRS complex peak is then detected with a simple slope-based peak detection algorithm. Figure 2g shows the average HR by both the sensors. Supplementary Fig. 2b shows the strong heartbeat modulated on the thorax NCS sensor while performing different breathing exercises, especially visible in the breath-hold periods, with the corresponding HR from both sensors in Supplementary Fig. 2e.

#### Breath-hold detection

This is based on estimating the time interval between two peaks at the end of inspiration, which consists of one exhalation and one inhalation duration. True detection is marked if a breath-hold period is larger than a fixed duration in the annotated breath-hold period, otherwise a missed detection is marked. To simulate CSA characterized by repeated interruptions in breathing during sleep^42^, we used our breath-hold data collected in supine and lateral recumbent postures, with participants performing two consecutive breath-holds in each posture, separated by normal breathing periods. For consistency, the same algorithm is used to estimate the numbers of pauses from both BIOPAC abdomen belt and NCS.

#### Paradoxical abdomen-thorax motion detection

Isovolumetric exercises lead to opposite slopes of thorax and abdomen respiration waveforms during abdomen contraction and relaxation periods, denoted by a negative product of instantaneous slopes, which are scaled to [−1, 1] using a ${\mathrm{tanh}}()$ function before estimating the product, then moving-averaged over a window of 1 s and thresholded ($< 0$) to give the detected instances. The ground-truth window is manually labeled, and a true detection is marked if it overlaps with the detected periods, otherwise, a missed detection is marked. As the slope sign is independent of waveform scaling, this detection does not require PTM calibration.

#### Data quality estimation

Different approaches are followed for respiration and heartbeat data quality estimation.

**Fig. 5:**
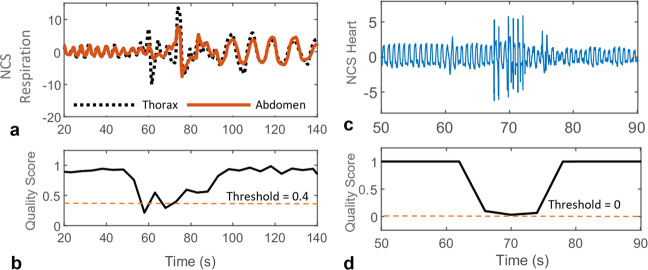
The quality score for respiration and heartbeat waveforms with motion artifact instance between 60–80 s. A fixed, empirically selected threshold is used to reject poor data, given by a low quality score. **a** Normalized NCS thorax and abdomen respiration waveforms. **b** Respiration quality score in the range [0,1], showing poor quality with a score less than the threshold (0.4). **c** Normalized filtered NCS heartbeat waveform modulated on the thorax sensor, with most of the artifact filtered out. **d** Quality score normalized by ${\mathrm{tanh}}()$ in the range [−1, +1]. A low threshold of 0 is selected, as the second-harmonic method used for heart rate estimate provides even higher motion tolerance.

(1) Respiration data quality: As thorax and abdomen sensors are independently placed, motion artifacts may be present differently, leading to a poor correlation between the two measurements. Also, data from NCS and BIOPAC chest belts can show dissimilar artifacts, with both or only either one of them showing the artifact. Thus, correlations between thorax and abdomen chest belts, and NCS and chest belts for both thorax and abdomen are calculated with an epoch duration of 5 s. Final data quality is taken as an absolute of the product of all these correlations, with higher values indicating better quality, as shown in Fig. 5a, b. A fixed threshold is empirically selected, below which the RR and RV estimates are discarded.

(2) Heartbeat data quality: As the NCS heartbeat waveform is only extracted from the thorax sensor, a different artifact detection algorithm is used, similar to that presented in our previous work^52^. An outlier detection algorithm based on one-class support vector machine (OCSVM)^53^ is implemented to detect artifacts in the filtered heartbeat waveform. To speed up signal processing, the motion detection algorithm is implemented on a window of 4 s. The algorithm is trained on the entire routine and a window is marked as an outlier if the ${\mathrm{tanh}}()$ normalized score is <0. Figure 5c, d show the filtered heartbeat waveform from the NCS thorax sensor and the corresponding score, respectively. For ECG waveforms, QRS complex peaks are less sensitive to noise, and thus no separate artifact detection is implemented. HR estimation is discarded if the window contained any poor-quality period.

### Reporting summary

Further information on research design is available in the Nature Research Reporting Summary linked to this article.

## Supplementary information

Supplementary Information

Reporting Summary

## Data Availability

The collected and analyzed data sets during the current study are available from the corresponding author upon request.

## Code availability

The LabVIEW code for data collection is available at https://github.com/EdwinKanLab/Respiration_Heart_Study_2019, and MATLAB codes for data processing are available from the corresponding author upon request.

## References

[1] Matheson EM, King DE, Everett CJ (2012). Healthy lifestyle habits and mortality in overweight and obese individuals. J. Am. Board Fam. Med..

[2] Sanchis-Gomar F (2015). Physical inactivity and low fitness deserve more attention to alter cancer risk and prognosis. Cancer Prev. Res..

[3] Detering KM, Hancock AD, Reade MC, Silvester W (2010). The impact of advance care planning on end of life care in elderly patients: randomised controlled trial. BMJ.

[4] Mitchell G (2017). Rapidly increasing end-of-life care needs: a timely warning. BMC Med..

[5] Rubio N (2017). Home monitoring of breathing rate in people with chronic obstructive pulmonary disease: observational study of feasibility, acceptability, and change after exacerbation. Int. J. Chron. Obstruct. Pulmon. Dis..

[6] Graham BL (2019). Standardization of spirometry 2019 update an official American Thoracic Society and European Respiratory Society technical statement. Am. J. Respir. Crit. Care Med..

[7] DePaso WJ, Winterbauer RH, Lusk JA, Dreis DP, Springmeyer SC (1991). Chronic dyspnea unexplained by history, physical examination, chest roentgenogram, and spirometry: analysis of a seven-year experience. Chest.

[8] Wanger J (2005). Standardisation of the measurement of lung volumes. Eur. Respir. J..

[9] Farré R, Montserrat JM, Navajas D (2004). Noninvasive monitoring of respiratory mechanics during sleep. Eur. Respir. J..

[10] Massaroni C (2019). Contact-based methods for measuring respiratory rate. Sensors.

[11] Rofail LM, Wong KKH, Unger G, Marks GB, Grunstein RR (2010). Comparison between a single-channel nasal airflow device and oximetry for the diagnosis of obstructive sleep apnea. Sleep.

[12] Brüllmann G, Thurnheer R, Bloch E (2010). Respiratory monitoring by inductive plethysmography in unrestrained subjects using position sensor-adjusted calibration. Respiration.

[13] Chu M (2019). Respiration rate and volume measurements using wearable strain sensors. NPJ Digit. Med..

[14] Tremper KK (1989). Pulse oximetry. Chest.

[15] Biswas D, Simoes-Capela N, Van Hoof C, Van Helleputte N (2019). Heart rate estimation from wrist-worn photoplethysmography: a review. IEEE Sens. J..

[16] Zheng Y (2014). Unobtrusive sensing and wearable devices for health informatics. IEEE Trans. Biomed. Eng..

[17] Inan OT (2015). Ballistocardiography and seismocardiography: a review of recent advances. IEEE J. Biomed. Heal. Inform..

[18] Taebi A, Solar B, Bomar A, Sandler R, Mansy H (2019). Recent advances in seismocardiography. Vibration.

[19] Xia Z, Member S, Shandhi MH, Member S (2018). Non-contact sensing of seismocardiogram. IEEE Sens. J..

[20] Pinheiro E, Postolache O, Girão P (2010). Theory and developments in an unobtrusive cardiovascular system representation: Ballistocardiography. Open Biomed. Eng. J..

[21] Kim CS (2016). Ballistocardiogram: Mechanism and potential for unobtrusive cardiovascular health monitoring. Sci. Rep..

[22] Sadek I, Seet E, Biswas J, Abdulrazak B, Mokhtari M (2018). Nonintrusive vital signs monitoring for sleep apnea patients: a preliminary study. IEEE Access.

[23] Pinheiro E, Postolache O, Girão P (2012). Study on ballistocardiogram acquisition in a moving wheelchair with embedded sensors. Metrol. Meas. Syst..

[24] *Hexoskin**Smart Shirts-cardiac, Respiratory, Sleep & Activity Metrics*, https://www.hexoskin.com/ (2020).

[25] Bansal C, Scott R, Stewart D, Cockerell CJ (2005). Decubitus ulcers: a review of the literature. Int. J. Dermatol..

[26] Zeevi T, Levy A, Brauner N, Gefen A (2018). Effects of ambient conditions on the risk of pressure injuries in bedridden patients—multi-physics modelling of microclimate. Int. Wound J..

[27] Reyes BA (2017). Tidal volume and instantaneous respiration rate estimation using a volumetric surrogate signal acquired via a smartphone camera. IEEE J. Biomed. Heal. Inform..

[28] Li C, Lubecke VM, Boric-Lubecke O, Lin J (2013). A review on recent advances in Doppler radar sensors for noncontact healthcare monitoring. IEEE Trans. Microw. Theory Tech..

[29] Naishadham K (2016). Estimation of cardiopulmonary parameters from ultra wideband radar measurements using the state space method. IEEE Trans. Biomed. Circuits Syst..

[30] Droitcour AD, Boric-Lubecke O, Kovacs GTA (2009). Signal-to-Noise ratio in Doppler radar system for heart and respiratory rate measurements. IEEE Trans. Microw. Theory Tech..

[31] Zito D (2011). SoC CMOS UWB pulse radar sensor for contactless respiratory rate monitoring. IEEE Trans. Biomed. Circuits Syst..

[32] Nguyen, P., Zhang, X., Halbower, A. & Vu, T. Continuous and fine-grained breathing volume monitoring from afar using wireless signals. In *IEEE INFOCOM**2016 - The 35th Annual IEEE International Conference on Computer Communications*, pp. 1–9 (IEEE, San Francisco, CA, 2016). 10.1109/INFOCOM.2016.7524402.

[33] Adib, F., Mao, H., Kabelac, Z., Katabi, D. & Miller, R. C. Smart homes that monitor breathing and heart rate. *In Proceedings of the 33rd Annual ACM Conference on Human Factors in Computing Systems (CHI ’15)*, pp 837–846 (Association for Computing Machinery, New York, NY, USA, 2015). 10.1145/2702123.2702200.

[34] Massagram W, Hafner N, Lubecke V, Boric-Lubecke O (2013). Tidal volume measurement through non-contact Doppler radar with DC reconstruction. IEEE Sens. J..

[35] Pfanner F, Allmendinger T, Flohr T, Kachelrieß M (2013). Modelling and simulation of a respiratory motion monitor using a continuous wave Doppler radar in near field. Proc. SPIE Med. Imag..

[36] Teichmann D, Kuhn A, Leonhardt S, Walter M (2014). The main shirt: a textile-integrated magnetic induction sensor array. Sensors.

[37] Hui X, Kan EC (2018). Monitoring vital signs over multiplexed radio by near-field coherent sensing. Nat. Electron..

[38] Hui, X., Sharma, P. & Kan, E. C. Microwave stethoscope for heart sound by near-field coherent sensing. In *EEE MTT-S International Microwave Symposium (IMS)*, pp. 365–368 (IEEE, Boston, MA, USA, 2019). 10.1109/MWSYM.2019.8700904.

[39] Li C, Cummings J, Lam J, Graves E, Wu W (2009). Radar remote monitoring of vital signs. IEEE Microw. Mag..

[40] Sharma, P., Hui, X. & Kan, E. C. A wearable RF sensor for monitoring respiratory patterns. In *41st Annual International Conference of the IEEE Engineering in Medicine and Biology Society (EMBC)*, pp. 1217–1223 (IEEE, Berlin, Germany, 2019). 10.1109/EMBC.2019.8857870. 10.1109/EMBC.2019.8857870 31946112

[41] Occupational Safety and Health Administration, United States Department of Labor. *OSHA safety and health topics: Radiofrequency and microwave radiation standards*, https://www.osha.gov/SLTC/radiofrequencyradiation/standards.html (2020).

[42] Muza RT (2015). Central sleep apnoea-a clinical review. J. Thorac. Dis..

[43] Konno K, Mead J (1967). Measurement of the separate volume changes of rib cage and abdomen during breathing. J. Appl. Physiol..

[44] Ambrosino N, Scano G (2004). Dyspnoea and its measurement. Rev. Lit. Arts Am..

[45] Campbell ML, Templin T, Walch J (2010). A respiratory distress observation scale for patients unable to self-report dyspnea. J. Palliat. Med..

[46] Gordon, P. H., Chen, R., Park, H. & Kan, E. C. Embroidered antenna characterization for passive UHF RFID tags. In *IEEE RFID Conf*., https://arxiv.org/abs/1710.02237 (2016).

[47] USRP B200mini. https://www.ettus.com/all-products/usrp-b200mini-board/ (2020).

[48] *Data acquisition and analysis system with AcqKnowledge for MP36R* (BIOPAC Systems, Inc., 2020). https://www.biopac.com/product/mp36r-systems/.

[49] Stoet G (2010). PsyToolkit: a software package for programming psychological experiments using Linux. Behav. Res. Methods.

[50] Stoet G (2017). PsyToolkit: a novel web-based method for running online questionnaires and reaction-time experiments. Teach. Psychol..

[51] Lu W (2006). A semi-automatic method for peak and valley detection in free-breathing respiratory waveforms. Med. Phys..

[52] Sharma, P. & Kan, E. C. Sleep scoring with a UHF RFID tag by near field coherent sensing. In *IEEE/MTT-S International Microwave Symposium - IMS*, pp. 1419–1422 (IEEE, Philadelphia, PA, 2018). 10.1109/MWSYM.2018.8439216.

[53] Scholkopf, B., Williamson, R., Smola, A., Shawe-Taylor, J. & Platt, J. Support vector method for novelty detection. In *Proceedings of the 12th International Conference on Neural Information Processing Systems (NIPS’99)*. pp. 582–588 (MIT Press, Cambridge, MA, USA, 1999).

