# Supplementary material for: Wearable radio-frequency sensing of respiratory rate, respiratory volume, and heart rate
Source: NPJ Digit Med. 2020 Jul 28;3:98. doi: 10.1038/s41746-020-0307-6 (PMC7387475; doi:10.1038/s41746-020-0307-6)
Supplement: Supplementary file 1 — Supplementary Information [file 41746_2020_307_MOESM1_ESM.pdf]

Supplementary Information

Wearable Radio-Frequency Sensing of Respiratory Rate, Respiratory Volume and Heart Rate

Pragya Sharma[1]*, Xiaonan Hui[1], Jianlin Zhou[1], Thomas B. Conroy[1] and Edwin C. Kan[1]

[1] School of Electrical and Computer Engineering, Cornell University, Ithaca, NY, USA
*e-mail: ps847@cornell.edu

Contents:

Supplementary Fig. 1. NCS heartbeat waveform and HR extraction 3

Supplementary Fig. 2. Examples of a 5-minute breathing protocol for one participant 4

Supplementary Fig. 3. Comparison of NCS and BIOPAC data 5

Supplementary Fig. 4. Breath-hold detection for two participants on instances of good and poor cases 6

Supplementary Fig. 5. Two representative cases for paradoxical abdomen-thorax motion detection 6

Supplementary Fig. 6. The effect of ambient motion on BIOPAC and NCS signals 7

Supplementary Fig. 7. The effect of hand motion on BIOPAC and NCS signals 7

Supplementary Fig. 8. The effect of arm motion on BIOPAC and NCS signals 8

Supplementary Fig. 9. Results of calibration consistency test 8

Supplementary Fig. 10. Studying the effect of simulated airway resistance variation 9

Supplementary Table 1. Correlation and B&A statistics for each participant 10

Supplementary Table 2. Average RV, RR and HR over 3 postures: supine, left lateral recumbent and sitting 10

Supplementary Table 3. RV calibration consistency test over 3 consecutive days 10

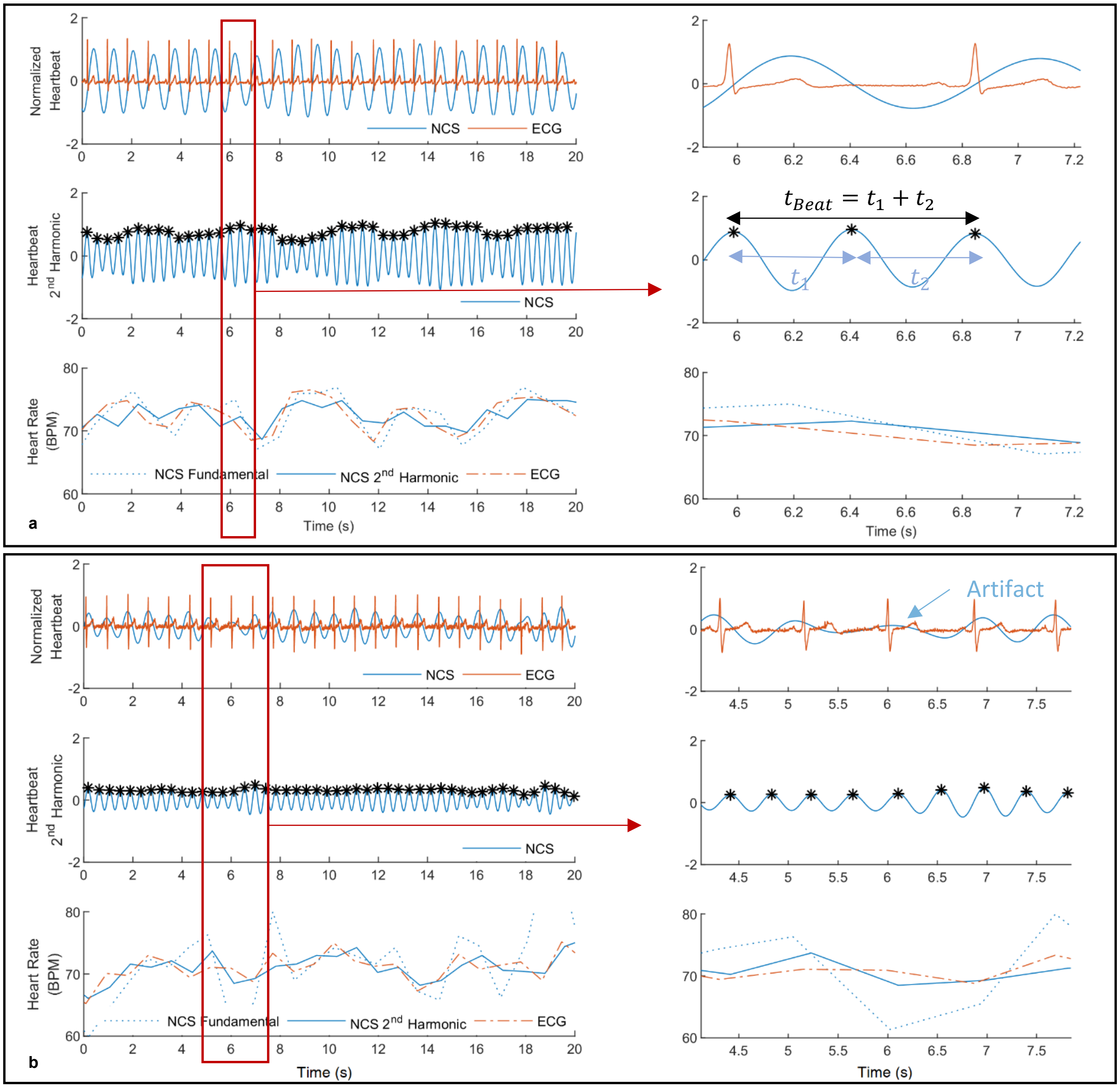

Supplementary Fig. 1. NCS heartbeat waveform and HR extraction. *Left:* Band-pass filtered NCS heartbeat waveforms and heart rate extraction from 2nd harmonic, showing instances without motion artifact (**a**) and with motion artifact (**b**). **a** Top figure shows ECG and filtered NCS heartbeat waveform from the thorax sensor. The middle figure shows 2nd harmonic of the NCS heartbeat waveform with detected peaks. The bottom figure shows the instantaneous heart rate, which is the inverse of each beat-to-beat interval, t_{Beat}, from fundamental NCS (dotted blue), harmonic NCS (solid blue) and ECG (dash-dotted orange). **b** The same heart rate extraction plots for another participant, showing periods of motion artifact at $t = 6$ s and 18 s, where the fundamental NCS heartbeat would have resulted in wrong heart rate estimation, but the 2nd harmonic is less affected. *Right:* Zoomed-in version of the left figures, showing t_{Beat} calculation from NCS 2nd harmonic as the sum of adjacent beat-to-beat intervals.

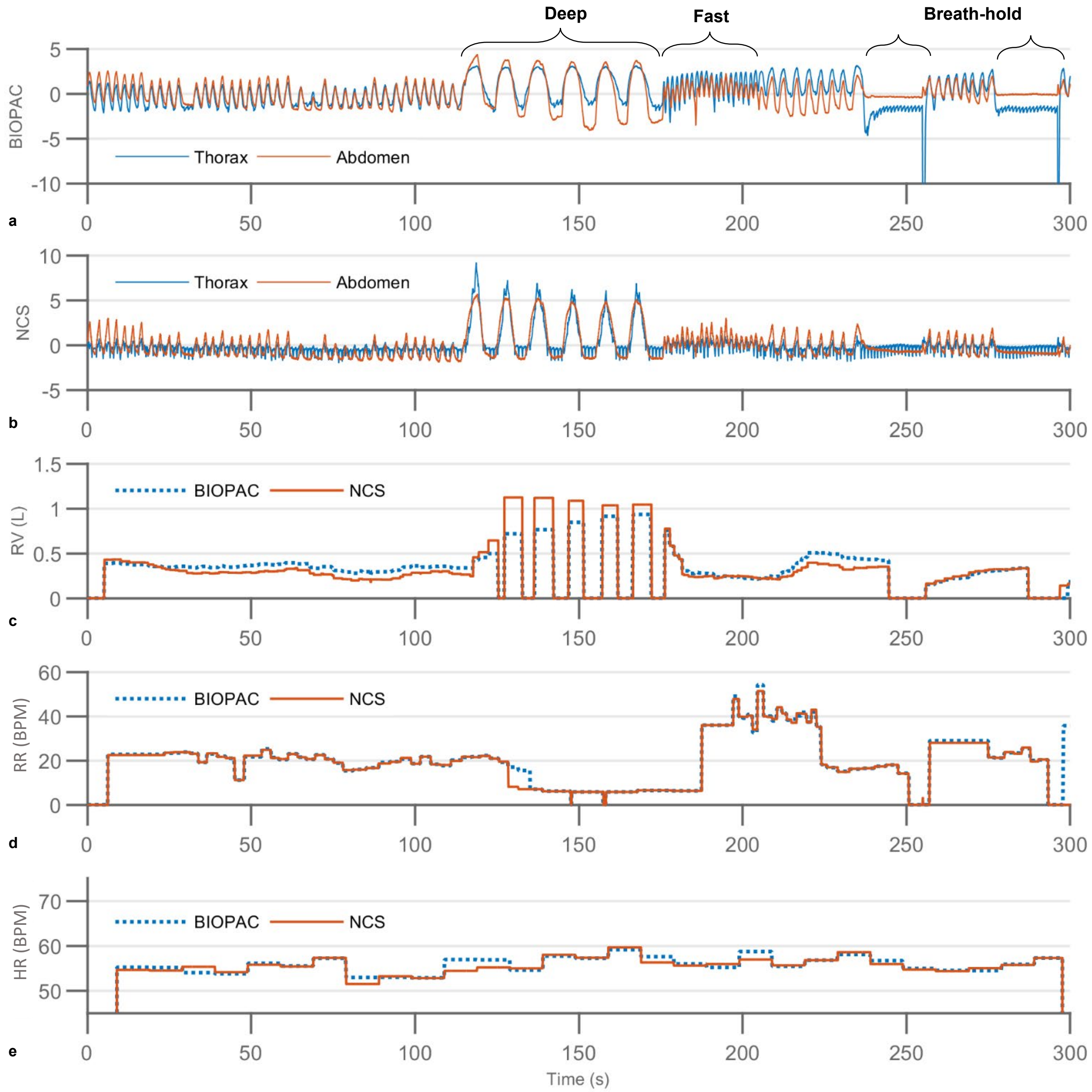

Supplementary Fig. 2. Examples of a 5-minute breathing protocol for one participant. The subject is in the supine posture while performing normal, deep, fast breathing and breath-hold. **a** Normalized BIOPAC chest-belt data at thorax and abdomen. During breath hold the thorax belt also shows a weak heartbeat motion. Different breathing periods are indicated here, with normal breathing in the unmarked sections. **b** Normalized NCS respiration data from thorax and abdomen sensors, as well as strong heartbeat on the thorax waveform. **c** RV during different breathing styles, based on the average volume exchanged in each inhalation and exhalation cycle over the past window containing at least two peaks, thus resulting in 0 estimates when the number of peaks is less than two (slow breathing). **d** RR estimation from NCS and BIOPAC clearly showing different breathing periods, with the normal RR around 20 BPM. **e** HR estimation from both sensors showing average resting HR in the range of 55 — 60 BPM.

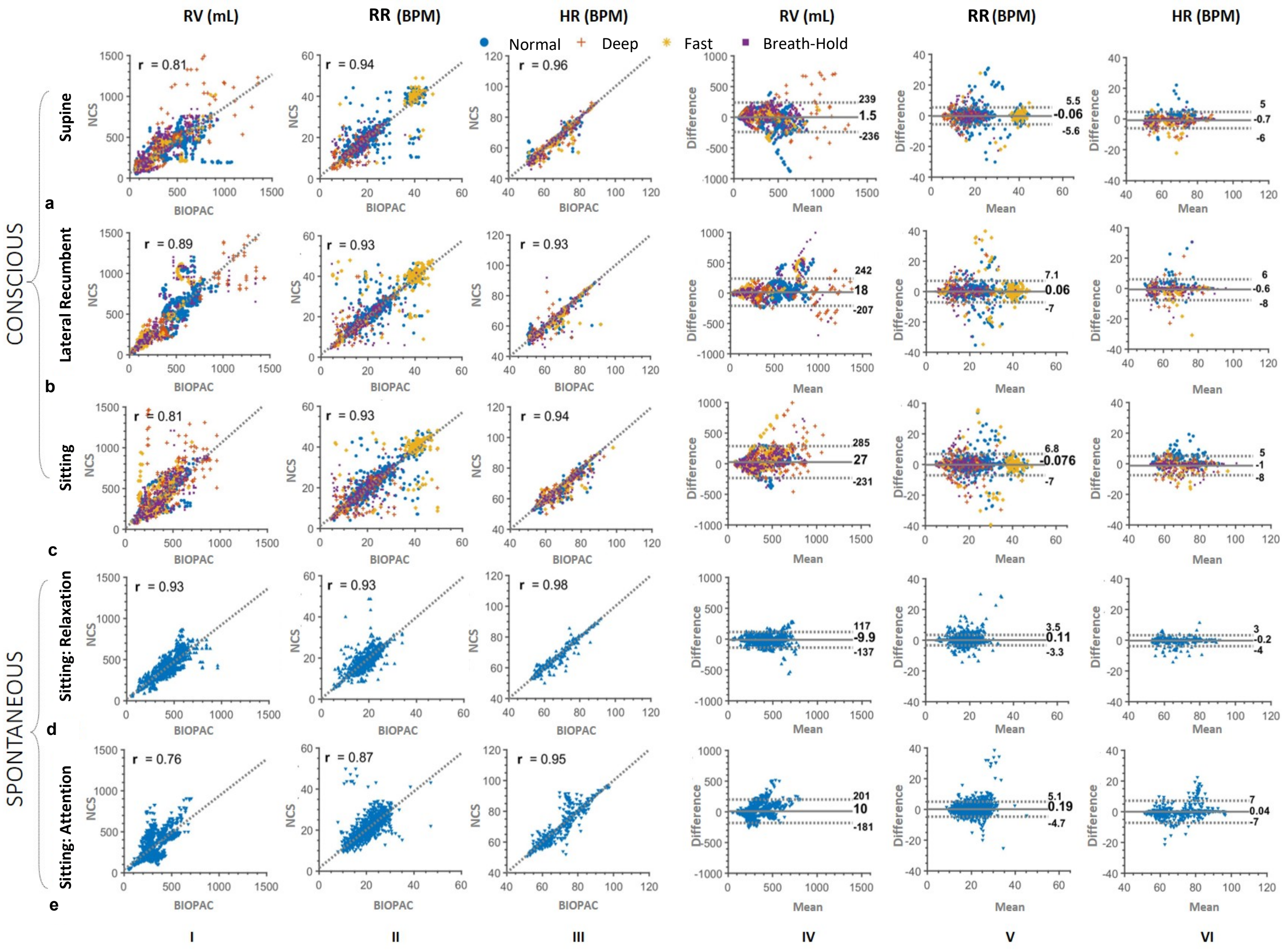

Supplementary Fig. 3. Comparison of NCS and BIOPAC data. Scatter (I – III) and $B\&A$ plots (IV – VI) between NCS and BIOPAC showing correlation coefficients (r), bias (m) and limits of agreement (LoA: $m \pm 1.96 \cdot \sigma$) for the RV, RR and HR across all routines. **a – c** The results across supine, lateral recumbent and sitting postures while following the breathing protocol of normal, deep, fast breathing and intermittent breath-hold. Different breathing protocol periods are indicated with a specific marker. **d, e** The spontaneous breathing protocol results in the relaxation and attention states with the participant sitting upright.

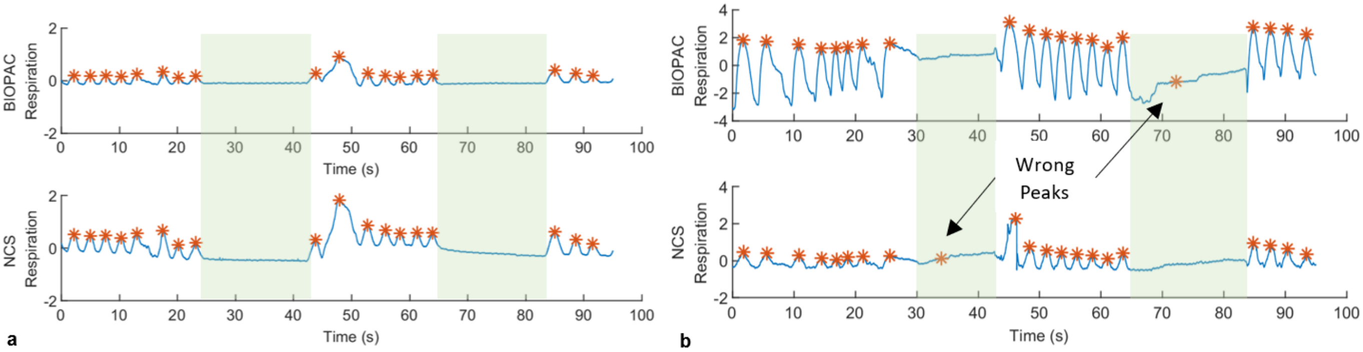

Supplementary Fig. 4. Breath-hold detection for two participants on instances of good and poor cases. Green shaded sections show manually annotated simulated apnea durations. The top and bottom figures show normalized BIOPAC and NCS abdomen respiration waveforms with detected peaks. The participant can perform the breath hold without any motion in **a**, leading to accurate detection. In **b**, wrong peaks are detected in both NCS and BIOPAC waveforms as shown, due to artifacts and peak detection limitations, as the participant is not able to maintain the breath hold without any motion. The motion coupling is different for the two sensors, as seen around $t = 68$ s, where BIOPAC shows some abdomen motion leading to wrong peak detection, but not NCS.

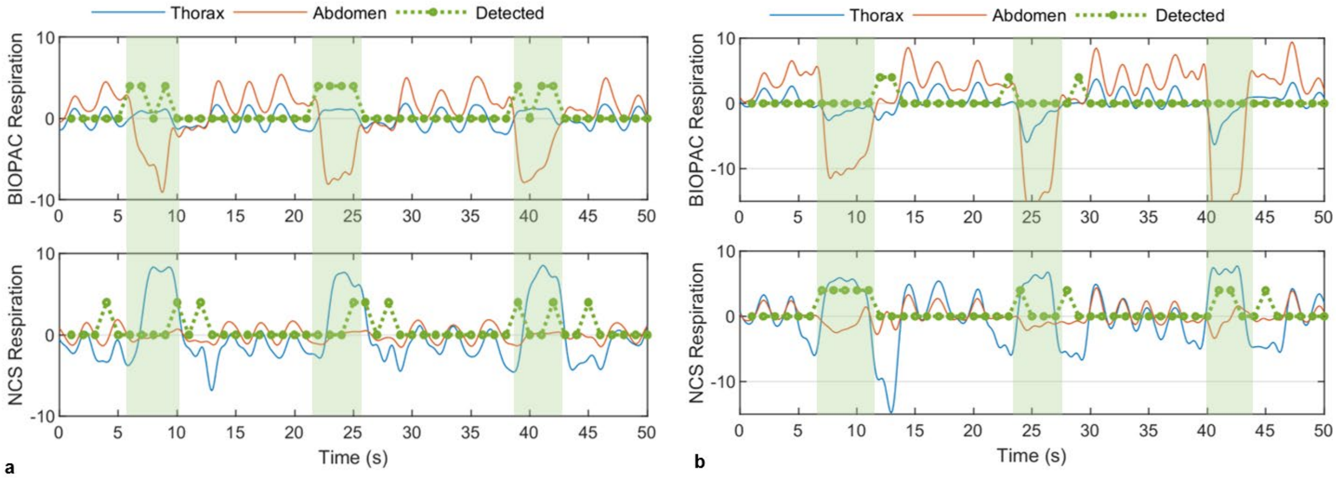

Supplementary Fig. 5. Two representative cases for paradoxical abdomen-thorax motion detection. Normalized respiratory waveforms from BIOPAC chest belts and filtered NCS are plotted in top and bottom figures, respectively. Intended instances are manually annotated by the green shaded areas. The detected periods are shown by the dotted green lines. True detection is marked if the annotated window overlaps with the observed instances. **a** The BIOPAC waveforms show clear paradoxical motion visually, as well as by the algorithm. NCS thorax and abdomen sensors do not show a complete paradoxical motion visually, but there are periods of opposite slope leading to detection of the second and third instances. Better abdomen sensor placement is required. **b** NCS waveforms show clear paradoxical motion visually, as well as by the algorithm. BIOPAC thorax belt do not show clear paradoxical motion in all three instances and requires better placement.

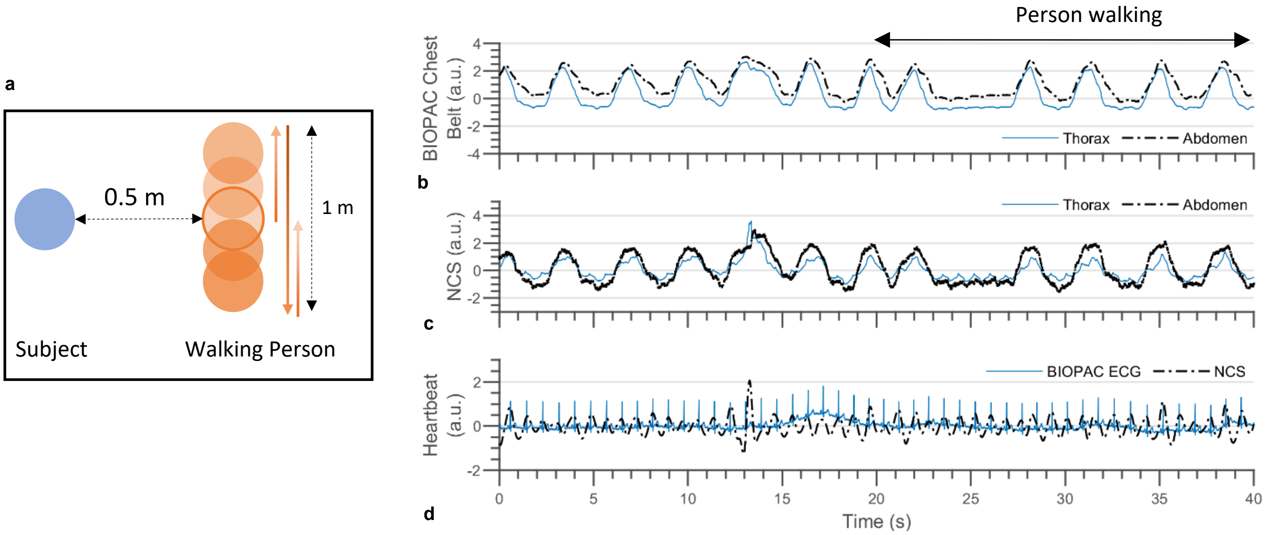

Supplementary Fig. 6. The effect of ambient motion on BIOPAC and NCS signals. Subject is seated at rest with a person standing nearby at a distance of 0.5 m. At $t = 20\ s$, the person starts walking forward and backward at a speed of 0.73 m/s as shown in **a**. **b** Normalized BIOPAC chest belt waveforms showing clear respiratory signal with no motion interference. **c** Normalized NCS waveforms showing both respiration and clear heartbeat without any motion interference. **d** Clear heartbeat signal is observed from both ECG and NCS without any interference.

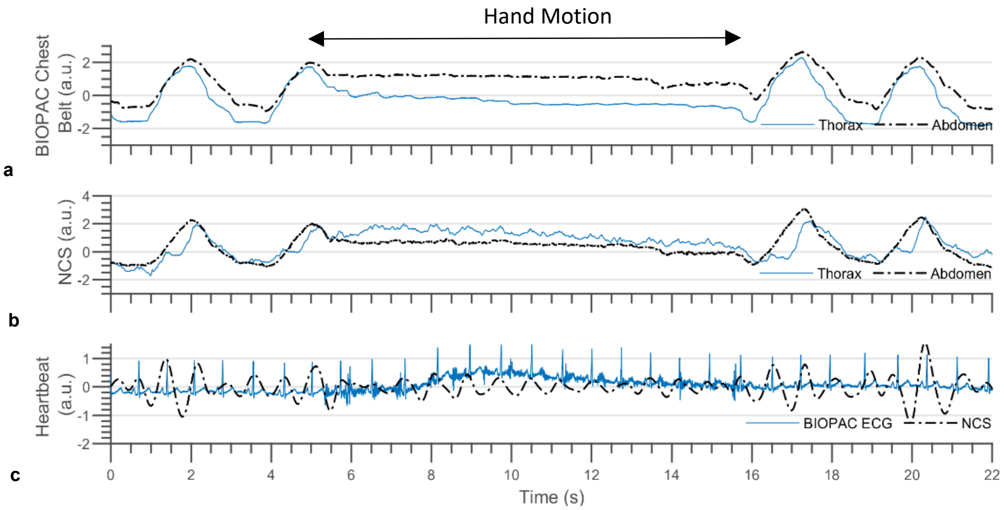

Supplementary Fig. 7. The effect of hand motion on BIOPAC and NCS signals. The subject is seated at rest with hands resting on thighs, performing normal breathing for the first 5 s. For the next 10 s, repeated instances of fist opening and closing are performed with the right hand, while holding breath. **a** Normalized BIOPAC chest-belt waveforms showing clear respiratory signals with no motion artifact. **b** Normalized NCS waveforms showing respiratory motion, with minimal motion artifact. **c** Heartbeat motion can be correctly extracted from NCS after further processing. Some interference is observed in the ECG waveform during the hand motion, but the R peaks can still be clearly seen.

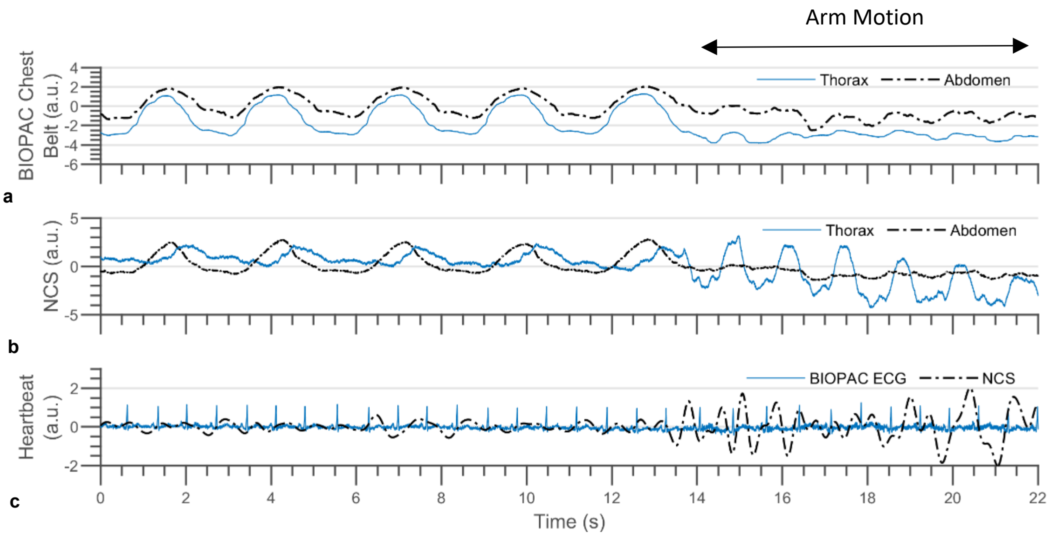

Supplementary Fig. 8. The effect of arm motion on BIOPAC and NCS signals. The subject is seated at rest with horizontal forearms in front of the body and upper arms in the vertical position. Forward arm swinging motion is performed starting at $\boldsymbol{t = 14\ s}$, with maximum angle of around $\mathbf{30}°$, while holding breath and keeping forearms horizontal. **a** Normalized BIOPAC chest-belt waveforms showing clear respiratory signals and some motion interference during the breath hold period with arm motion. **b** Normalized NCS waveforms showing respiratory motion without any interference to the abdomen sensor, but arm motion is captured in the thorax sensor. **c** The heartbeat signal from NCS is interfered due to the arm swing and HR can only be possibly extracted from the harmonic.

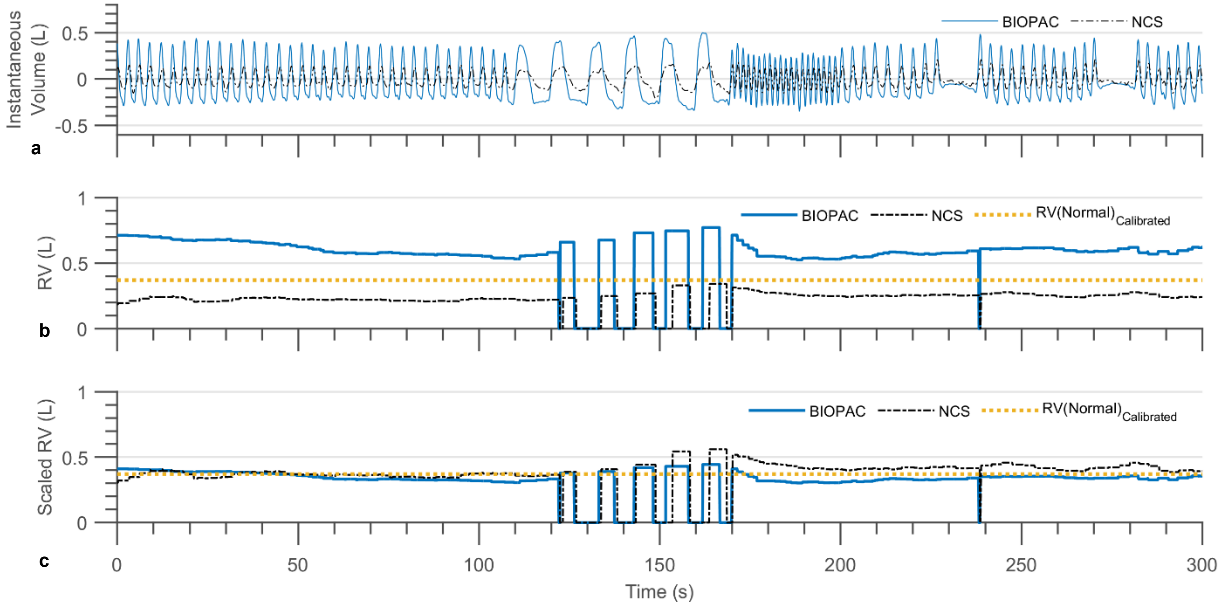

Supplementary Fig. 9. Results of calibration consistency test: using RV calibration from day-1 on day-2 data, both collected in sitting posture. Subject is performing voluntary breathing exercises in the routine. **a** Calibrated instantaneous volume from BIOPAC and NCS. **b** Estimated RV from the top plot with BIOPAC overestimating and NCS underestimating the expected $\mathrm{RV(Normal)}_{\mathrm{Calibration}} = 0.37$ L. **c** Scaled RV estimation that corrects the error based on a normal breathing section.

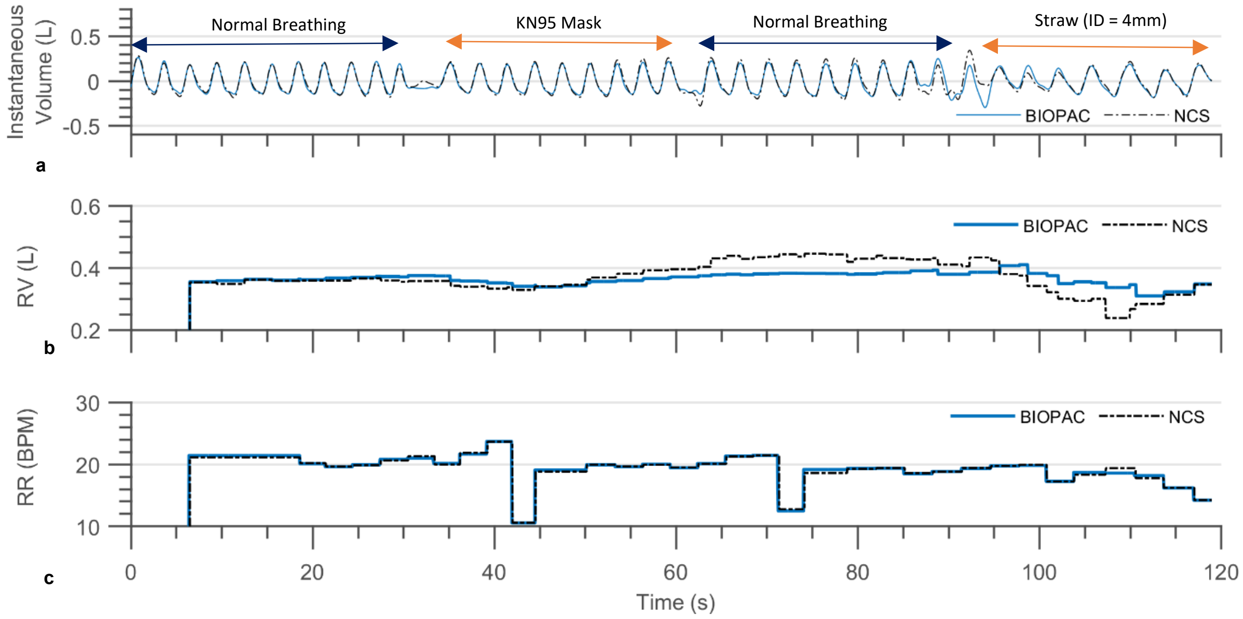

Supplementary Fig. 10. Studying the effect of simulated airway resistance variation. Subject is sitting upright while performing normal breathing. Two external resistances are introduced: 1) KN95 mask, while breathing normally through nose, and 2) straw with an inner diameter (ID) of 4 mm, only breathing through mouth. **a** & **b** plots show **c**alibrated instantaneous volume and derived RV estimates respectively, **c** shows corresponding RR. The response to mask is nearly not distinguishable from the normal breathing from both RV and RR estimates, slight dips around $t = 45$ s and 70 s are due to the breath holds during the transition periods. Breathing through straw shows decreased RV in the beginning, while maintaining same RR, the following increase in RV is associated with decreased RR, showing the increased respiratory effort required due to the resistance offered by the narrow straw. While both NCS and BIOPAC can capture these trends, NCS appears to be more sensitive to the RV changes.

Supplementary Table 1. Correlation and $B\&A$ statistics for each participant

Measurement		Subjects																		
		1	2	3	4	5	6	8	9	10	11	12	13	14	15	16	17	18	19	20
**RV (mL)**	$\mathbf{r}$	0.6	0.85	0.68	0.73	0.71	0.80	0.45	0.80	0.20	0.84	0.97	0.90	0.76	0.71	0.86	0.72	0.92	0.43	0.82
	$\boldsymbol{m}$	40	4	210	12	-8	58	21	-57	88	-12	-29	-9	-41	14	21	-3	1	0	-46
	$\boldsymbol{\sigma}$	125	41	195	39	102	93	96	123	179	84	36	83	64	55	105	188	28	99	140
**RR (BPM)**	$\mathbf{r}$	0.85	0.91	0.87	0.92	0.90	1	0.93	0.90	0.97	0.91	0.91	0.91	0.97	0.78	0.82	0.91	0.90	1.00	0.93
	$\boldsymbol{m}$	-0.07	0.20	0.88	-0.27	0.17	-0.02	-0.27	0.01	-0.16	0.32	-0.46	-0.13	-0.20	0.39	0.53	0.50	0.27	0.01	-0.29
	$\boldsymbol{\sigma}$	3.08	2.50	5.38	2.86	2.64	0.68	2.45	3.36	2.09	2.80	3.85	2.49	1.99	4.26	4.84	2.63	2.87	0.45	3.43
**HR (BPM)**	$\mathbf{r}$	0.97	0.45	0.36	0.57	0.98	0.87	0.33	0.92	0.81	0.87	0.61	0.91	0.92	0.81	0.74	0.72	0.80	0.86	0.92
	$\boldsymbol{m}$	-0.20	-1.06	-3.96	-0.36	-0.20	-0.06	0.08	0.20	-0.41	-0.39	-0.90	-1.01	0.10	-0.13	-0.27	-1.06	-0.12	-0.33	-0.11
	$\boldsymbol{\sigma}$	1.26	4.42	6.08	2.92	1.19	2.20	7.44	1.13	2.15	2.40	2.43	2.79	2.10	2.31	1.83	2.58	1.00	1.63	1.42

Table showing Pearson's correlation coefficient (r), and mean bias (m) and standard deviation (σ) of the difference of NCS and BIOPAC estimates ($B\&A$ statistics) for each subject across all the postures and breathing styles. Subject 7 is not shown here, as at least half of the data is discarded due to poor quality, resulting in a small data set for any statistical analysis.

Supplementary Table 2. Average RV, RR and HR over 3 postures: supine, left lateral recumbent and sitting

**Posture**	**RV (mL)**	**RR (BPM)**	**HR (BPM)**
**Supine**	329 ± 200	17.3 ± 8.5	65.3 ± 9.9
**Left Lateral Recumbent**	263 ± 210	19.5 ± 9.5	63.8 ± 9.7
**Sitting**	317 ± 179	19 ± 9.7	67.6 ± 9.3

Table showing mean $(m) \pm$ standard deviation (σ) of the RV, RR and HR statistics over different postures. The data is collected over all 20 participants following the same voluntary breathing exercise routine of normal, deep, and fast breathing, as well as breath holds. Average RV is observed to be highest in the supine posture, followed by sitting and lateral recumbent, while RR has the opposite trend. HR is highest in the sitting posture, followed by supine and lateral recumbent postures. The standard deviation likely demonstrates variation introduced from different breathing exercises.

Supplementary Table 3. RV calibration consistency test over 3 consecutive days

**1. Day $\boldsymbol{i}$**	**2. $\mathbf{RV(Normal)_{Calibration}}$**	**3. $\mathbf{RV(Normal)_{Routine}}$** (Calibration: Day **1**)	**4. Scaling Factor, $\boldsymbol{SF}$** (Calibration: Day **1**)
$\boldsymbol{i = 1}$	$\mathrm{RV}_{PTM} = 0.37\ L$	$\mathrm{RV}_{NCS} = 0.39\ L$, $\mathrm{RV}_{BIOPAC} = 0.35\ L$	$SF_{NCS} = 0.95, SF_{BIOPAC} = 1.06$
$\boldsymbol{i = 2}$	$\mathrm{RV}_{PTM} = 0.43\ L$	$\mathrm{RV}_{NCS} = 0.22\ L$, $\mathrm{RV}_{BIOPAC} = 0.65\ L$	$SF_{NCS} = 1.68, SF_{BIOPAC} = 0.57$
$\boldsymbol{i = 3}$	$\mathrm{RV}_{PTM} = 0.43\ L$	$\mathrm{RV}_{NCS} = 0.24\ L$, $\mathrm{RV}_{BIOPAC} = 0.60\ L$	$SF_{NCS} = 1.54, SF_{BIOPAC} = 0.62$

Table displaying the normal breathing RV calibration and scaling factor consistency over three days of testing on the same subject. Each day consisted of a calibration period immediately followed by a breathing routine, performed while sitting upright. **1.** The day of the measurements. **2.** The normal RV estimated from the PTM during the calibration routine. All three RV estimates are close to each other, within a range of 0.06 L. **3.** The normal breathing RV for NCS and BIOPAC during the breathing exercise routine, derived using calibration from day 1 for all three days. **4.** The calculated scaling factor to accurately map the routine's normal RV to normal RV of day 1 calibration. Little scaling is required to correct RV when calibrated immediately before the routine, resulting in scaling factors close to 1 on day-1. Variations in sensor placement and coupling strength result in scaling factors other than 1 to correct the normal RV on the next two days.
